# Isolation of In Vitro Osteoblastic-Derived Matrix Vesicles by Ultracentrifugation and Cell-Free Mineralization Assay

**DOI:** 10.21769/BioProtoc.5258

**Published:** 2025-04-05

**Authors:** Irshad A. Sheikh, Pawel R. Kiela, Fayez K. Ghishan

**Affiliations:** Department of Pediatrics, University of Arizona, Tucson, AZ, USA

**Keywords:** Extracellular vesicles, Matrix vesicles, MC3T3-E1 subclone 4 preosteoblast cell line, Ultracentrifugation, Mineralization, Nanoparticle tracking analysis, Energy dispersive spectroscopy (EDS) analysis in scanning electron microscopy (SEM)

## Abstract

Matrix vesicles (MVs) represent a heterogeneous group of spherical membrane-bound extracellular vesicles in the range of 100–200 nm in diameter secreted by mineralizing osteoblasts. The initial synthesis of the amorphous calcium phosphate occurs within the confines of the intracellular MVs, which are capable of transporting P_i_ and Ca^2+^ into the MV lumen. Thus, understanding the initial process of MV-mediated mineralization is critical in developing better therapeutic strategies for various bone-related disorders such as osteoporosis and addressing ectopic calcification of soft tissues. Although various techniques and commercially available kits are now available for isolating MVs, isolating a pure population of MVs is challenging mainly because of their variable size and lack of consensus protein markers. This ultracentrifugation-based protocol ensures high purity of isolated MVs by removing other contaminated extracellular vesicles and cellular debris through sequential centrifugation steps but also allows downstream functional mineralization assays of the isolated MVs.

Key features

• Simple and rapid high-quality isolation of MVs from in vitro culture of mineralizing osteoblasts by ultracentrifugation.

• Use of isolated MVs for various functional assays such as mineralization efficacy.

• Cell-free mineralization assay to determine intrinsic mineralization efficacy of the isolated MVs under desired experimental conditions.

## Graphical overview


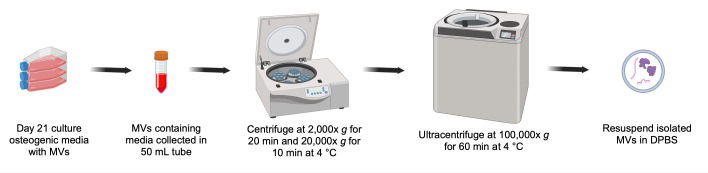


**Overall scheme for isolation of matrix vesicles (MVs) from osteogenic media**

## Background

Matrix vesicles (MVs) are extracellular membrane-bound nanovesicles enriched with various enzymes that transport proteins to facilitate the synthesis of hydroxyapatite (HA) and transmembrane flux of Ca^2+^ and PO_4_^3-^ to their lumen. The initial amorphous HA forms on the inner leaflet of MVs and acts as the primary nucleation center of mature HA crystals, which continue to grow to form calcified nodules [1,2].

Based on recent studies, MVs are not only involved in mineralization but also carry nucleic acid and protein cargo within their lumen that participates in autocrine and paracrine exchange of intracellular or intercellular signals [3,4]. Because of this functional role of MVs in signal transduction and the initial nucleation centers for bone formation, there is an urgent need for a better understanding of the functional physiology of MVs and how their functions are affected by various inflammatory conditions such as chronic inflammation and osteoporosis [5].

As more researchers continue to decipher and acknowledge various physiological roles of MVs, many different methods of MVs isolation have been developed, each having its own pros and cons as well as differences in purity and required isolation time [6,7]. Some of the most widely used methods include ultracentrifugation, precipitation kits, size exclusion chromatography, ultrafiltration, and magnetic bead technologies [8]. Choosing a suitable isolation method for MVs depends on the volume and type of starting material, the scientific query to be addressed, and subsequent analysis of the isolated MVs. Moreover, the protein and nucleic acid composition of MVs is influenced by the method of isolation protocol used. Hence, it is crucial to determine the most appropriate isolation protocol in order to ensure accurate interpretation and data reproducibility.

Here, we describe the most commonly used ultracentrifugation-based protocol for MV isolation, which we recently used to unravel the role of NCX3 in facilitating Ca^2+^ entry within the MVs during the initial mineralization process [9]. This method of MV isolation offers the advantage of isolating native, unmodified MVs from culture media with relative ease [10]. The isolated MVs exhibit high purity and retain their intrinsic mineralization ability, features that can be leveraged to investigate the mineralizing properties of isolated MVs under desired experimental conditions in a cell-free environment. The potential drawbacks of the method include the relatively large scale of osteoblast culture, required access to an ultracentrifuge, and possible co-isolation of contaminants as this method is based on different sedimentation rates of particles that differ in size and density.

## Materials and reagents

**Biological materials**

1. MC3T3-E1 subclone 4 cell line, obtained from American Type Culture Collection (Manassas, VA; ATCC #CRL-2593). Cells should be grown to 70%–80% confluency in growth media (see Recipe 3) and maintained in a humidified environment at 37 °C in the presence of 5% CO_2_ in a cell culture incubator

**Reagents**

1. MEM Alpha (Gibco, catalog number: A10490-01)

2. Ascorbic acid (Sigma-Aldrich, catalog number: A4403)

3. Exosome-depleted FBS (Gibco, catalog number: A2720801)

4. Penicillin-streptomycin (100×) (Invitrogen, catalog number: 15140122)

5. Trypsin-EDTA (0.25%), phenol red (Gibco, catalog number: 25200072)

6. Dulbecco’s phosphate-buffered saline (DPBS) (Gibco, catalog number: 14190144)

7. Sodium phosphate dibasic (Na_2_HPO_4_) (Sigma, catalog number: S9763)

8. Pierce BCA Protein Assay kit (Thermo Scientific, catalog number: 23227)

9. Radioimmunoprecipitation assay (RIPA) lysis buffer (EMD Millipore, catalog number: 20-188)

10. Protease inhibitor cocktail (Sigma, catalog number: P8340)

11. HCl 37% (Acros Organics, catalog number: 124630010)

12. Acetic acid, glacial (Sigma, catalog number: 695092)

13. Calcium colorimetric assay (Sigma, catalog number: MAK022-1KT)

14. Bovine serum albumin (BSA) (Thermo Scientific Chemicals, catalog number: 9048-46-8)

15. Sterile water (Fisher Scientific, catalog number: SH3119101)

**Solutions**

1. Ascorbic acid solution (see Recipes)

2. Na_2_HPO_4_ solution (see Recipes)

3. Growth media (see Recipes)

4. Osteogenic media (see Recipes)

5. 0.6 N HCl (see Recipes)

**Recipes**

**1. Ascorbic acid solution (1,000× stock)**

**Table:** 

Reagent	Final concentration	Weight/Volume
Ascorbic acid	50 mg/mL	50 mg
Sterile water		1 mL

Store at -20 °C protected from light for up to 2–4 weeks.

**2. Na_2_HPO_4_ solution (1 M)**

**Table:** 

Reagent	Final concentration	Weight/Volume
Na_2_HPO_4_	1 M	4.26 g
Sterile water		30 mL

Store at room temperature for up to 2–3 months.

**3. Growth media**

**Table:** 

Reagent	Final concentration	Volume
MEM Alpha	1×	44.5 mL
FBS	10% v/v	5 mL
Penicillin-streptomycin (100×)		500 μL
Total		50 mL

Store at 4 °C for up to 2 weeks. FBS was heat inactivated at 55 °C for 30 min prior to use.

**4. Osteogenic media**

**Table:** 

Reagent	Final concentration	Volume
MEM Alpha	1×	44.35 mL
FBS	10% v/v	5 mL
Penicillin-streptomycin (100×)	1×	500 μL
Ascorbic acid (Recipe 1)	50 μg/mL	50 μL
Na_2_HPO_4_ (Recipe 2)	3 mM	150 μL
Total		50 mL

Always prepare fresh and protect from light.

**5. 0.6 N HCl**

a. Measure 4.97 mL of 37% HCl using a 5 mL pipette in a 100 mL beaker.

b. Add distilled water until the volume reaches 100 mL.

c. Mix thoroughly with a magnetic stirring bar inside the beaker.

**Laboratory supplies**

1. 1.5 mL microcentrifuge tubes (USA Scientific, catalog number: 1415-2500)

2. 15 mL conical tubes (Sarstedt, catalog number: 62.553.205)

3. 50 mL conical tubes (Sarstedt, catalog number: 62.547.205)

4. Serological pipettes 10 mL (Sarstedt, catalog number: 86.1254.001)

5. Serological pipettes 25 mL (Sarstedt, catalog number: 86.1685.001)

6. Tissue culture flask, 150 cm^2^, canted neck, vented cap (Corning, catalog number: 430825)

7. Tissue culture flask, 25 cm^2^ filter (Greiner, catalog number: 690175)

8. 0.45 μm sterile syringe filters (Sarstedt, catalog number: 83.1826)

9. Open-top thin-wall ultracentrifuge tube, 38.5 mL capacity (Beckman Coulter, catalog number: 344058)

10. TEM Grids G300 (SPI Supplies, catalog number: 2030C-XA) for electron microscopy

11. 22 mm collagen-coated glass coverslip (Sigma, catalog number: CLS354089)

12. Forceps (Sigma, catalog number: F4017)

13. 100 mL beaker (Fisher Scientific, catalog number: FB100100)

14. Magnetic stirring bar (Fisher Scientific, catalog number: 14-512-126)

15. 12-well cell culture plates (Fisher Scientific, catalog number: 07-200-82)

16. Large-orifice pipette tips (Fisher Scientific, catalog number: 02-707-134)

## Equipment

1. CO_2_ cell culture incubator (e.g., Thermo Scientific, catalog number: 51033557)

2. Ultra-centrifuge with a swinging bucket rotor [e.g., Beckman Coulter, model: Optima LE-80K with SW32 Ti swinging-bucket rotor (Beckman Coulter, catalog number: 369694)]

3. Electron microscope (Hitachi, model: HF5000) equipped with an adapter for X-ray microanalysis, energy dispersive spectrometer (EDS, Oxford Instruments)

4. Nanosight LM10-HS instrument (Malvern Instruments, Worcestershire, UK), equipped with an sCMOS camera and 638 nm laser

## Software and datasets

1. GraphPad Prism version 9.5.1 (GraphPad Software, San Diego, CA, USA)

2. BioRender (https://BioRender.com, Toronto, ON, Canada)

## Procedure

**A. Acquisition and culture of mouse MC3T3-E1 osteoblast progenitor cell line**

1. A mineralizing subclone 4 of MC3T3-E1 osteoblast progenitor cell line (ATCC # CRL-2593) was selected because it exhibits high osteoblast differentiation and mineralization after growth in media containing ascorbic acid and inorganic phosphate.

2. Culture MC3T3-E1 cells (passages 8–21) in a T25 flask with 5 mL of growth media at 37 °C with 5% CO_2_.

3. Under standard growth and maintenance conditions, replace the culture medium every two days and subculture cells at a 1:4 ratio with 0.25% trypsin at 80%–90% confluency.

4. To induce osteogenic differentiation and mineralization, seed 2.5–3 × 10^6^ MC3T3-E1 cells in 30 mL of growth media in a T150 flask with regular media change every two days. Grow until 80%–90% confluency is reached. At that point, switch media from growth to osteogenic media and culture for an additional 21 days with media changes every three days.

5. Collect osteogenic media after 21 days of culture for MV isolation and place on ice.

*Note: If not subjected to MV isolation immediately, the conditioned osteogenic media can be stored at -80 °C for several weeks without compromising the quality and quantity of MVs.*

**B. Isolation of matrix vesicles by ultracentrifugation**

*Note: All subsequent steps should be conducted at 4 °C and samples should be kept on ice.*

1. Centrifuge the osteogenic media at 2,000× *g* for 20 min at 4 °C to remove any cell debris.

2. Collect the supernatant into a fresh 50 mL tube and centrifuge at 20,000× *g* for 30 min at 4 °C to remove large-sized vesicles or apoptotic bodies.

*Note: Alternatively, filter the supernatant using a 0.45 μm syringe filter.*

3. Transfer the supernatant into pre-chilled ultracentrifuge polypropylene tubes.

4. Centrifuge at 100,000× *g* for 1 h at 4 °C for MV isolation.

5. Gently decant the supernatant and suspend the MV pellet in 50 μL of ice-cold 1× DPBS by pipetting up and down.

*Note: The pellet may be difficult to see clearly. In such a case, it is recommended to carefully remove the supernatant, leaving up to 10 μL behind.*

6. The resulting MV suspension in 20 μL of DPBS is ready for downstream analysis or can be stored in a 1.7 mL microcentrifuge tube at -80 °C for later use.

*Note: Average MV yield from 30 mL of culture media of one T150 flask ranges between 35 and 50 μg of protein.*

**C. Characterization of matrix vesicles**

1. Quantification of total MV protein by BCA analysis:

a. Prepare BSA dilutions (see Table 1) in 1.7 mL microcentrifuge tubes to obtain a BCA standard curve.

**Table 1.** BCA standard curve preparation

Albumin standard dilution (mg/mL)	Albumin stock solution (2 mg/mL) (μL)	H_2_O (μL)
2.00	200	-
1.00	100	100
0.5	50	150
0.25	25	175
0.125	12.5	187.5
0.0625	6.25	193.75

b. Add 80 μL of 1× RIPA buffer and 2 μL of protease inhibitor cocktail to 20 μL of MVs suspended in 1× DPBS and mix thoroughly.

i. Keep the MVs suspended in lysis solution for 1 h for protein enrichment.

ii. Centrifuge at 14,000× *g* for 20 min.

iii. Collect the supernatant in a 1.7 mL microcentrifuge tube.

c. Pipette 10 μL of BSA standards and MV sample in triplicates in the same 96-well plate.

d. Add 200 μL of BCA working solution to each well.

e. Incubate the plate at 37 °C for 30 min and evaluate the protein concentration at OD 562 nm with a microplate reader.

f. Calculate the protein concentration by plotting the BCA standard curve (see Table 2).

**Table 2.** BCA analysis. Total MV protein calculation performed by BCA analysis. Six BSA standards in serial dilutions were used and average absorbance was calculated at 562 nm.

Sample	OD 1	OD 2	OD 3	Average OD	Concentration
BSA S1 (2,000 μg/mL)	1.04	0.98	0.99	1.003	
BSA S2 (1,000 μg/mL)	0.536	0.544	0.516	0.532	
BSA S3 (500 μg/mL)	0.282	0.269	0.281	0.277	
BSA S4 (250 μg/mL)	0.152	0.167	0.151	0.157	
BSA S5 (125 μg/mL)	0.099	0.085	0.096	0.093	
BSA S6 (62.5 μg/mL)	0.039	0.029	0.37	0.035	
BSA S6 (0 μg/mL)	0.009	0.010	0.007	0.008	
MV sample	0.222	0.211	0.223	0.219	375 μg/mL

**Figure 1.**
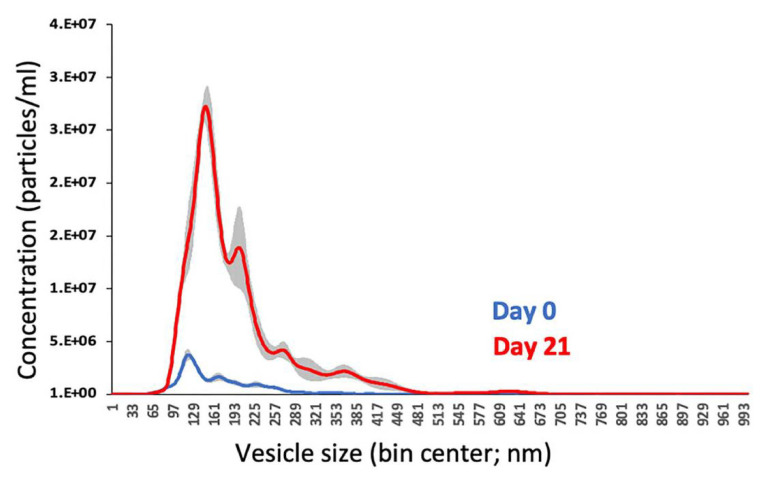
Nanoparticle tracking analysis (NTA) of isolated matrix vesicles (MVs). NTA histogram showing the size distribution of MVs isolated from osteogenic media of MC3T3-E1 cell culture on Day 0 (confluency; blue curve) and Day 21 of post-confluent osteogenic culture (red curve). Shaded grey areas represent SEM.

2. Size evaluation and quantification by nanoparticle tracking analysis (NTA): NTA is an advanced technique utilized to measure the size distribution of MVs. This method tracks the Brownian motion of individual vesicles within a laser-illuminated sample, providing precise particle size measurements in real time [11].

a. Dilute the isolated MV sample in 1 mL of DPBS and vortex for 15 s.

b. Using a needleless syringe, slowly inject the MV solution into the chamber of the particle size analyzer, taking care to avoid bubbles.

c. Ensure there are approximately 30–50 particles in the field of view to achieve accurate concentration and size measurements (Figure 1).

**Figure 2.**
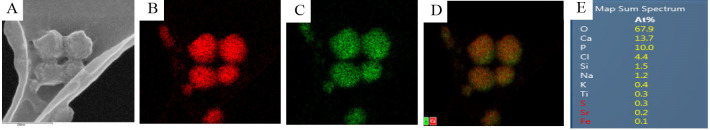
Scanning electron microscopy coupled with energy dispersive spectroscopy (SEM–EDS) analysis of the isolated matrix vesicles (MVs). (A) Representative SEM image of the isolated MVs deposited on the copper grid. Representative imaging of calcium (B, red) and phosphorus (C, green) and their overlay (D) using SEM-EDS. (E) Spectral map of isolated MV composition represented as atomic % of individual elements detected. In mature hydroxyapatite (HA), the approximate Ca/P ratio is 1.6, though the composition of amorphous calcium phosphate in MVs can vary, leading to the observed value of 1.37. Scale bar = 250 nm.

3. (Optional) Examine the calcium-to-phosphate (Ca/P) ratio within the isolated MVs by scanning electron microscopy coupled with energy dispersive spectroscopy (SEM–EDS). SEM–EDS is a powerful technique employed for the identification and quantification of Ca^2+^ and P content by detecting the characteristic X-rays emitted by calcium and phosphorus atoms when the MVs are exposed to a focused electron beam [12].

a. Deposit 10 μL of MV suspension prepared in sterile DPBS on lacey carbon-coated 300 mesh copper grids.

b. Air dry the MV sample for 30 min at room temperature and observe under 200 kV HF5000 transmission electron microscope equipped with an adapter for X-ray microanalysis energy dispersive spectrometer.

c. The images and EDS mapping were taken in scanning transmission electron microscopy (STEM) mode (Figure 2).

d. Spectral and compositional analysis is performed, and data is expressed as a percentage of the sum of the known elements detected within the MVs to indicate the content of Ca and P (as atomic %).

**Figure 3.**
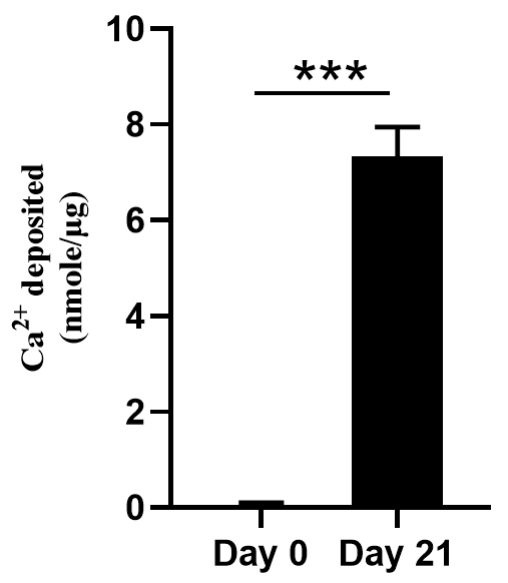
Calcification assay of the isolated matrix vesicles (MVs). Cell-free in vitro MV-collagen calcification assay with MVs isolated from Day 0 and Day 21 culture media of MC3T3-E1 osteoblasts.

4. Examine the mineralization efficacy of isolated MVs by cell-free calcification assay.

a. Place the collagen-coated glass coverslips in the wells of a 12-well cell culture plate.

b. Add 25 μg of MV (based on protein assay) to each coverslip in the presence of 200 μL of osteogenic media and ensure it is evenly distributed across the coverslip surface.

c. Incubate the coverslips at 37 °C for 72 h in a CO_2_ incubator.

d. After 72 h, gently remove (decant) the osteogenic media from the coverslips without disturbing the MV-collagen matrix.

e. Gently wash the coverslips once with 1× DPBS and add 250 μL of 0.6 N HCl to each well to cover the coverslips completely. Incubate at room temperature for 24 h to decalcify the matrix.

g. After 24 h of decalcification, collect the 250 μL of HCl supernatant carefully without disturbing the coverslips.

h. Measure the calcium content in the HCl supernatant using a colorimetric Ca^2+^ assay kit as per the manufacturer’s instructions (Figure 3).

## Data analysis

All the experiments were performed at least in triplicates, and MVs isolated from growth media at Day 0 were used as appropriate controls. The results obtained from the characterization techniques are summarized as follows:

BCA analysis: The total protein concentration of MVs isolated from 30 mL of osteogenic medium was 375 μg/mL (Table 2).

NTA: The isolated MVs had an average size of 166.9 nm, which falls within the expected size range for MVs (100–200 nm), as depicted in Figure 1.

SEM–EDS: SEM imaging revealed a homogeneous population of MVs with spherical morphology and uniform size. The EDS analysis identified a Ca/P ratio of 1.37, consistent with the mineral composition of MVs, as shown in Figure 2.

These results confirm the successful isolation and characterization of MVs, providing quantitative and qualitative evidence of their integrity and purity.

## Validation of protocol

This protocol has been validated and implemented to generate Figure 5 from the following research article:

• Sheikh et al. [9]. The Na^+^/Ca^2+^ exchanger NCX3 mediates Ca^2+^ entry into matrix vesicles to facilitate initial steps of mineralization in osteoblasts. *Journal of Extracellular Vesicles*. 13(6): e12450. doi: 10.1002/jev2.12450.

## General notes and troubleshooting

The culture media volumes used were 5 mL for T25 flasks and 30 mL for T150 flasks. To reduce waste, growth and osteogenic media were prepared in 50 mL conical tubes in small batches of 50 mL based on experimental requirements, since the ascorbic acid in osteogenic media is susceptible to oxidation and requires fresh preparation.

**Troubleshooting**

Due to their small size, MVs can aggregate after the ultracentrifugation or isolation process. Adding 0.1% BSA to storage buffers and using pipette tips with larger diameters can help prevent MVs from sticking to surfaces and aggregating.
